# A Large Stone Within a Ureteroceles: A Diagnostic Pitfall and the Utility of Holmium Laser Deroofing as a Viable Surgical Option

**DOI:** 10.1089/cren.2017.0139

**Published:** 2018-02-01

**Authors:** Hanna Jamil El-Khoury, Hau Choong Aw, Daniel Gilbourd, Madalena Ian-Pim Liu

**Affiliations:** Department of Urology, Casey Hospital, Monash Health, Berwick, VIC, Australia.

**Keywords:** ureteroceles, ureteral stones, holmium laser

## Abstract

We describe a case of a partial unilateral duplex system and ureterocele containing a 4 cm stone in a 66-year-old woman who presented with renal colic. Cystoscopic stone removal and deroofing of the ureterocele were performed and a ureteral stent was placed for a total of 6 weeks. Our case is unique as it highlighted the diagnostic pitfalls of ureteroceles, especially when obscured by a large calculus. We also described the use of a Holmium laser to simultaneously incise the ureterocele and fragment the calculus.

## Case Report

**FIG. 1.**
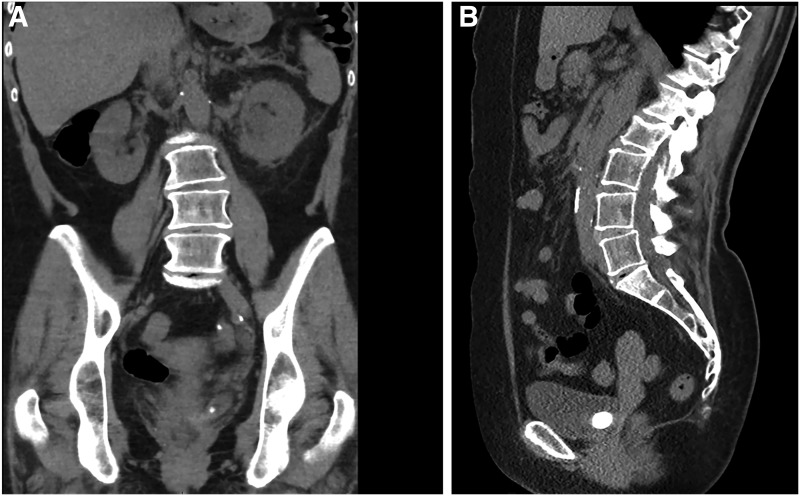
**(A)** Noncontrast CT abdomen, sagittal section illustrating a large stone at the vesicoureteral junction. **(B)** Coronal section of the same CT scan illustrating part of the stone within the ureterocele.

A 66-year-old woman presented to the emergency department with severe left loin to groin pain, nausea, vomiting for 24 hours duration. She denied any infective or systemic symptoms. She denied any history of stone disease, recurrent urinary tract infection, or childhood developmental issues. Physical examination revealed left loin and groin tenderness with voluntary guarding but no rigidity. Urine showed microhematuria and proteinuria, and culture was negative for infection. Her estimated glomerular filtration rate (eGFR) was 82 on presentation (normal >90). A noncontrast CT of the renal system showed a large obstructing 4 cm stone at the vesicoureteral junction with marked hydronephrosis (Fig. 1).

**FIG. 2.**
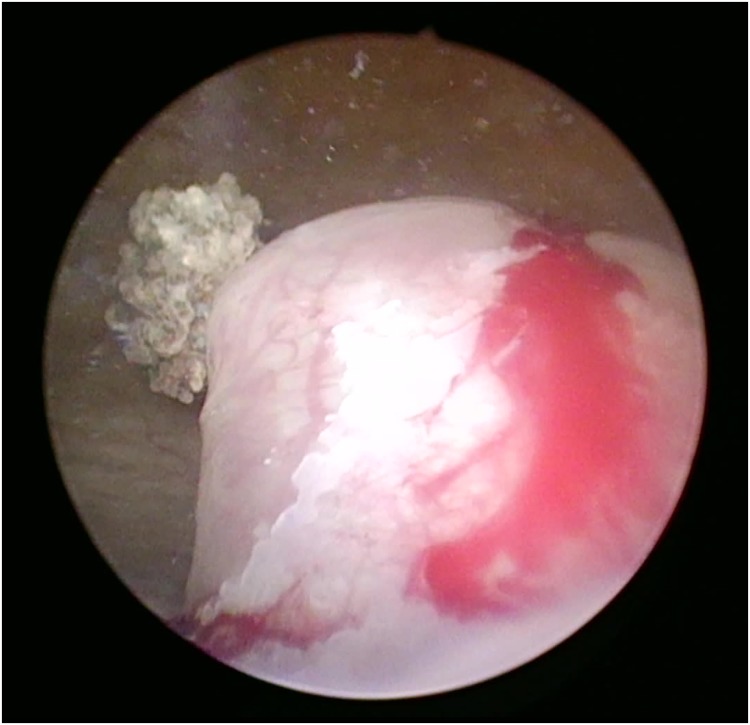
Intraoperative cystoscopic image showing the large ureteral stone pointing out of the ureteral orifice within a ureterocele.

**FIG. 3.**
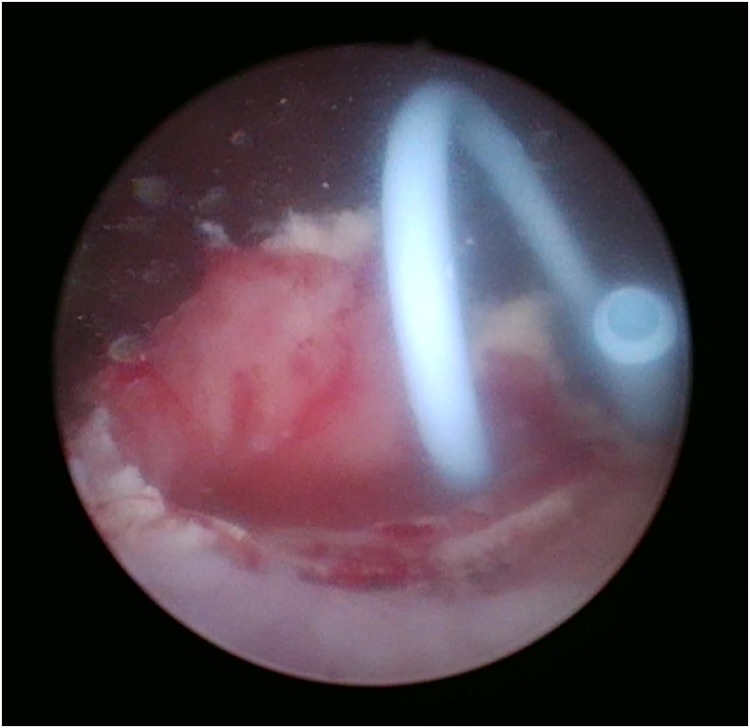
Intraoperative cystoscopic image postlaser and removal of the ureteral stone with the ureteral stent in place.

A cystoscopy was performed, which revealed a large stone within a ureterocele (Fig. 2). Deroofing of the ureterocele was performed in an L-shaped configuration using a holmium laser, similar to those used for a holmium enucleation of the prostate procedure (2 J at 25 Hz × 50 W). The stone was then segmented and extracted. A left ureteral stent was inserted (Fig. 3). The postoperative period was uneventful, and the patient was discharged home day 1 postoperation after voiding postremoval of urethral catheter. The ureteral stone measured ∼4 cm.

**FIG. 4.**
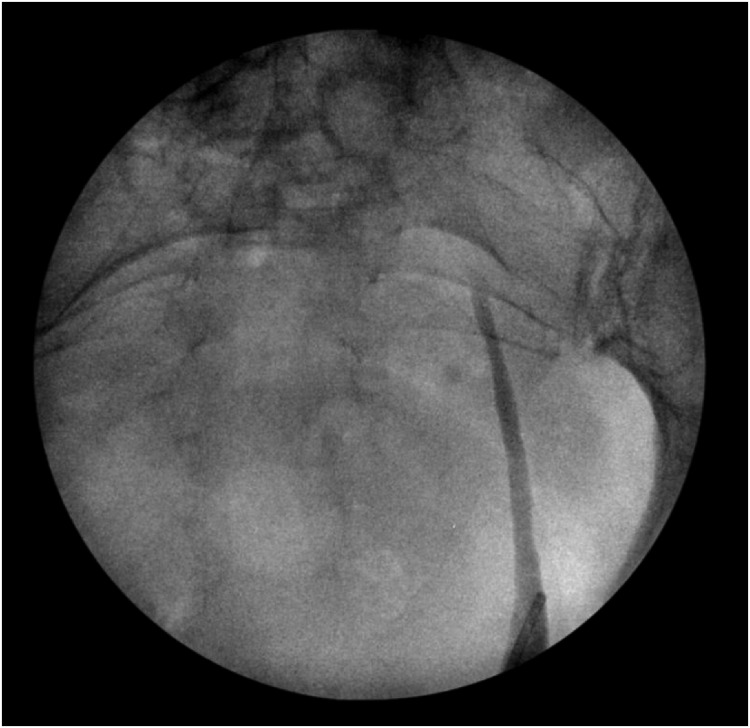
Retrograde pyelogram performed 6 weeks postderoofing of ureterocele illustrating no hydronephrosis or filling defects.

Approximately 6 weeks after the initial operation, the patient was brought back for a rigid cystoscopy and removal of the ureteral stent. At the time of this operation, it was noted that the patient had an open ureteral orifice with preservation of the ureteral sphincter. A retrograde pyelogram was performed, which illustrated no evidence of obstruction or hydronephrosis (Fig. 4). Her eGFR had returned to normal (>90). A renal tract ultrasound was performed 3 months postoperatively, which did not demonstrate hydronephrosis.

## Discussion

A ureterocele is defined as a cystic dilatation of terminal ureter with associated tissue defect in the urinary bladder. It may be associated with variable degree bladder muscle defect and renal parenchymal abnormality. The incidence of ureterocele is estimated to be 1 in 4000 live births.^1^

The commonly accepted theory behind ureterocele formation is the obstruction of ureteral orifice during embryogenesis, with incomplete dissolution of the Chwalla membrane.^1^ Approximately 10% of ureteroceles are bilateral. They occur most frequently in females with a 4:1 ratio and in Caucasians.^1^

Ureteroceles are divided into those that are completely inside the bladder, intravesical, or those that extend through the urethra or bladder neck, extravesical, and can also prolapse through the sphincter and cecoureterocele.^2^ Many patients remain asymptomatic, however, some present with recurrent urinary tract infection and severe pyelonephritis, frequency, nocturia, and severe renal colic from an obstructed calculus within the ureterocele.^3^ Asymptomatic patients require no treatment, however, those who present with symptoms eventually benefit from surgical incision of the blind-ending pouch.

The incidence of stones within a ureterocele, according to the literature, is between 5% and 40%.^2^ Case reports and literature on this condition are rare and management varies between centers and geographical origins.

A number of surgical techniques have been described in the literature. Endoscopic management of ureterocele has been gaining popularity. In current literature, transverse incisions of the ureterocele with monopolar diathermy have been described.^4^ Other cases have been described by utilizing shockwave lithotripsy initially, followed by transurethral ureterocele puncture.^4^ However, endoscopic puncture of the ureterocele has been illustrated to have high reoperation rates, ranging from 7% to 25%, many of which are performed as open surgery.^4^ Recently greenlight laser and holmium laser have been utilized for incisions of ureteroceles.

There are many pitfalls in the diagnosis of a ureterocele and our case highlighted these challenges. Classically, ultrasound of a well-distended bladder and ureterocele may reveal a thin sac overlying the ureteral orifice. Traditional intravenous pyelogram may also reveal a cystic dilatation of the distal ureter, known as the “cobra-head sign.” However, with the increasing use of CT, the use of intravenous pyelogram has become less common in the evaluation of stone disease. As illustrated in our case, the presence of a large calculus in the ureterocele may obscure the ureterocele, and as such, it may be suspected but can only be confirmed at the time of cystoscopy.

Extreme care has to be emphasized before incising ureteroceles. The ureterocele and ureter have to be clearly outlined with careful retrograde pyelograms. This is especially true in complete duplex ureters, as the position of the ureteral opening may be ectopic, and could be traversing the external sphincter. Inadequate anatomy outlining will result in inadvertent injury to the sphincter during incision, rendering the patient incontinent.

We believe our case to be the first in English literature to describe the use of holmium laser to deroof a ureterocele in an L-shaped configuration, without disruption of the underlying detrusor muscle. The incision commencing on the visible stone at the ureteral orifice and cutting onto the calculus protects the detrusor from injury. This enabled us to utilize the laser to breakup the stone into two to three pieces and safely remove the stones (Fig. 3). A retrograde pyelogram should be performed after stone removal to ensure that all stones are removed, and we recommend a ureteral stent placed for a period of 6 weeks postoperatively, to ensure adequate drainage of the kidney while allowing the distal ureter to heal. At such time, a retrograde pyelogram should be performed at the removal of the ureteral stent to ensure resolution of the obstruction.

Our method will help reduce the reoperation rates seen when utilizing conventional ureterocele punctures and incisions as described above, while being able to safely and quickly remove the obstructed system in a minimally invasive way. Laser incision has been shown to reduce scaring and strictures of the urothelium. A review of the literature reveals that utilization of holmium laser is not novel, and several small studies have been demonstrated to show decreased rates of vesicoureteral reflux in the postoperative period. However, the use of our technique and method will further help decrease rates of vesicoureteral reflux as it aims to preserve the underlying detrusor muscle, while maintaining the natural distal ureteral sphincter. It may be used in young adults with similar presentations, allowing for a minimally invasive technique without the high rates of ureteral reflux seen with conventional diathermy methods and standard transverse incisions.

### Learning Points

• Ureteroceles are rare, but should be considered in patients with recurrent urinary tract infections not responding to conventional therapy.
• Ureteroceles can become obstructed with large stones and cause renal colic once they cause complete ureter occlusion.
• Large distal calculus may mask a ureterocele on imaging, and high index of suspicion is required, especially when the stone size well exceeds the normal caliber of the ureter.
• Adequate outlining of the ureters and identification of an ectopic orifice will reduce the risk of inadvertent injury to the urethral sphincter.
• Holmium laser deroofing in an L-shaped configuration is a safe and minimally invasive method for treating ureteroceles with stones and a good alternative to monopolar diathermy.

## Patient Consent

Patient consent was obtained for this study.

## Abbreviations Used

CT: computed tomography
eGFR: estimated glomerular filtration rate
